# Editorial: Feature Representation and Learning Methods With Applications in Protein Secondary Structure

**DOI:** 10.3389/fbioe.2021.748722

**Published:** 2021-09-09

**Authors:** Ni Yan, Zhibin Lv, Wenjing Hong, Xue Xu

**Affiliations:** ^1^ School of Medicine, Wuhan University of Science and Technology, Wuhan, China; ^2^ Department of Medical Instruments and Information, College of Biomedical Engineering, Sichuan University, Chengdu, China; ^3^ State Key Laboratory of Physical Chemistry of Solid Surfaces, iChEM, Xiamen University, Xiamen, China

**Keywords:** protein secondary structure, structure prediction, deep learning, machine learning, feature extraction

In recent years, the rise of machine learning methods, especially deep learning, had greatly promoted the development of prediction of protein secondary structures. Such methods could not only make better use of exponentially growing massive protein sequence data, but were also able to automatically mine complex and latent patterns hidden in the data. Although significant progress had been made, we still faced challenges how to predict protein secondary structures directly from protein sequences with improved accuracy.

There were 11 articles published in the special issue *Feature Representation and Learning Methods With Applications in Protein Secondary Structure*. The authors here described computer methods and techniques for protein secondary structure predictions. Also, they presented and discussed latest algorithms development in feature extraction, dimension reduction, unbalanced classification, etc. The papers provided good references to those new to the field as well as experienced researchers.

Guo et al. established a model to classify thermophilic proteins and non-thermophilic proteins based on sequences. After feature extraction by iFeature, MRMD2.0 was applied for feature selection and dimension reduction, and LIBSVM was used to obtain the optimal parameters of the model and established the prediction model. Compared with LMT, Logistic, Random Forest, BayesNet, REPTree, J48, the prediction rate of this model was the highest (SE: 95.85%, SP: 96.22%, ACC: 96.02%).

Li et al. constructed a model to identify antioxidant proteins based on a support vector machine based method, Vote9. Sequence features were extracted by using reduced amino acid compositions and the optimal g-gap dipeptide compositions from nine optimal individual models.

Gu et al. distinguished GPCRs and non-GPCRs with CTDC extraction and MRMD2.0 dimension-reduction. The authors found different methods of feature extraction and the same method of dimensionality reduction had different effects on distinguishing GPCRs and non-GPCRs. The correct classification rate of five independent test sets was 90.64, 90.37, 88.04, 93.28, and 95.73%, with an average rate of 91.61 ± 2.96%.

Jing and Li used amino acid composition, dipeptide composition, position-specific score matrix auto-covariance, and Auto-covariance average chemical shift to predict cell wall lytic enzymes. SMOTE was used to counter the imbalanced data classification problems, and F-score algorithm was used to remove redundant or irrelevant features. ACC was 99.19% with jackknife test.

Chen et al. proposed a novel computational model for lncRNA-protein interaction relationship prediction based on machine learning methods. A method for representing the topological feature information of the network of lncRNA-protein interaction was proposed. Protein evolutionary information, protein CTD sequence information features, lncRNA sequence mutual information features, and lncRNA expression profile information were extracted, and the recursive feature elimination algorithm was used to optimize feature vectors. The obtained optimized feature vectors were fed into SVM to predict lncRNA-protein interactions. This method was experimentally compared with six excellent lncRNA-protein prediction algorithms, and experimental results showed that our proposed method achieves the best performance values in AUPR (74.39%) and F1 score (65.91%).

Li et al. used a total of 12 feature extraction methods when predicted anticancer peptides. After eight times of dimension reduction by MRMD2.0, they established a 19-dimensional feature model based on anticancer peptide sequences, which had lower dimension and better performance (ACC: 92.15–92.73%, SE: 85.5–87.7%, SP: 96.1–97.1%, MCC: 83.7–84.9%, F1 score: 92.1–92.7%) than some existing methods.

Wang et al. developed a bioinformatics tool called prPred for the prediction of plant resistance proteins that combines CKSAAP and CKSAAGP features based on SVM. Experimental results showed that the accuracy, precision, sensitivity, specificity, F1-score, MCC, and AUC of prPred were 0.935, 1.000, 0.806, 1.000, 0.893, 0.857, and 0.948, respectively, on an independent test set. The predictive and analytical results demonstrated that the constructed model was an efficient predictor to distinguish R proteins from non-R proteins.

Cai et al. established a comprehensive weight model SDN2GO based on protein sequence, protein domain content and known protein-protein interaction network. Compared with NetGO, DeepGO and the classic BLAST method, the authors’ results showed that SDN2GO achieved the maximum F-max value (36.1–56.1%) of each sub ontology of GO.

Liu et al. established a deep learning-based predictor TMPSS to predict the secondary structure and topological structure of α-helical TMPs. The TMPSS applied a deep learning network that included grouped multi-scale CNN (Convolutional Neural Network) and stacked attention-enhanced BiLSTM (Bidirectional Long Short-Term Memory) layers to capture local and global context. Based on the multi-task learning method, the prediction performance was improved and the amount of calculation was reduced by considering the interaction between different protein properties.

Yallapragada et al. established a game-based molecular visualization tool PePblock Builder VR-AN. Different from traditional sequence-based protein designs and fragment-based splicing, pepblockbuilder-VR provided a building block environment for the construction of complex structures, which provided users with a unique visual structure construction experience. In addition, Pepblock Builder VR worked as an independent and VR-based application and provided us with a good platform for teaching.

Lyu et al. established a reductive deep learning model MLPRNN to predict either 3-state or 8-state protein secondary structures, which had the same prediction accuracy as DeepCNF, MUFOLD-SS, BGRUCB, CRRNN and DNSS2.

The 11 papers in this research topic covered only a small part of the computer methods and techniques used to predict protein secondary structure. We hope more and more researchers will devote their time and effort into this field to predict the secondary structure of proteins more quickly, simply and accurately.

